# The impact of general/visceral obesity on completion of mesorectum and perioperative outcomes of laparoscopic TME for rectal cancer: A STARD-compliant article

**DOI:** 10.1097/MD.0000000000004462

**Published:** 2016-09-09

**Authors:** Bingchen Chen, Yuanchuan Zhang, Shuang Zhao, Tinghan Yang, Qingbin Wu, Chengwu Jin, Yazhou He, Ziqiang Wang

**Affiliations:** a The Surgical Department of Coloproctology, Zhejiang Provincial People's Hospital; b Department of General Surgery, The Third People's Hospital of Chengdu; c Department of Gastrointestinal Surgery, West China Hospital.

**Keywords:** completion of mesorectum, laparoscopic rectal surgery, surgical quality and difficulty, visceral fat area, visceral obesity

## Abstract

To evaluate the impact of visceral obesity on laparoscopic total mesorectal excision (TME) and decide the best index to reflect completion of mesorectum and perioperative outcomes.

Patients with rectal cancer who underwent laparoscopic TME were enrolled. The data including body mass index (BMI), visceral fat area (VFA), visceral fat area/body surface area (VFA/BSA), mesorectum fat ratio (MFR), pelvic fat area (PFA), pelvic fat ratio (PFR), completion of mesorectum, and other perioperative outcomes were collected. Data were analyzed.

A total of 322 patients were enrolled between 2011 and 2014. There was no significantly difference between the BMI groups on completion of mesorectum and other outcomes (*P* ≥ 0.05). However, in VFA groups, completion of mesorectum (*P* = 0.002), operative time (*P* = 0.02), and incision length (*P* = 0.02) were significantly different. In VFA/BSA groups, completion of mesorectum (*P* = 0.002) and incision length (*P* = 0.009) were significantly different. When MFR was equal to 0.48, completion of mesorectum (*P* = 0.002), operative time (*P* = 0.001), incision length (*P* = 0.03), and blood loss (*P* = 0.04) were significantly different between the 2 groups. In PFA and PFR groups, there was no significantly difference (*P* ≥ 0.05). After the analysis of logistic regression, only VFA was the risk factor of incomplete mesorectum excision.

BMI does not reflect the impact of obesity on laparoscopic rectal surgery. VFA is a better index in predicting the influence of visceral obesity on surgical quality and difficulty of laparoscopic rectal surgery than VFA/BSA and MFR.

## Abstract 2

Supplemental Digital Content is available in the text

## 1 Introduction

Reducing the rate of local recurrence after curative resection of rectum has always been the top issue in the rectal cancer management. Although total mesorectal excision (TME) has been developed and widely used, the rate of local recurrence remains at 4% to 10%.^[1]^ One of the key factors to prevent local recurrence is the complete excision of total mesorectum.^[2,3]^ However, TME could be extremely difficult to achieve due to excessive amount of intraabdominal fat, narrow pelvic, or lack of experience of surgeon.^[4–6]^ According to the standard proposed by Quirke et al,^[7]^ the completion of mesorectum was reported ranged from 10% to 90%. Therefore, evaluating risk factors of incomplete excision of mesorectum, such as general/visceral obesity, could be of value in tailoring individual treatment plan.

The aim of the present study is to evaluate the impact of general/visceral obesity on laparoscopic TME and decide the best index to reflect the completion of mesorectum and perioperative outcomes.

## 2 Materials and methods

This retrospective study was approved by the institutional review board at our institution, and the need to obtain informed consent was waived. From July 2011 to April 2014, consecutive patients with preoperative diagnosis of rectal cancer who underwent rectal surgery by the same medical team at the Department of Gastrointestinal Surgery were enrolled in this study.

### 2.1 Inclusion criteria

1. Pathologically confirmed rectal cancer after colonoscopy;
2. Laparoscopic procedure;
3. Complete data of preoperative computed tomography (CT), perioperative outcomes, and postoperative pathology.

### 2.2 Exclusion criteria

1. Transanal resection;
2. Incomplete data of preoperative CT, perioperative outcomes, and postoperative pathology;
3. Palliative resection;
4. Open procedure or conversion to open procedure.

### 2.3 Definition of obesity

**Figure 1:**
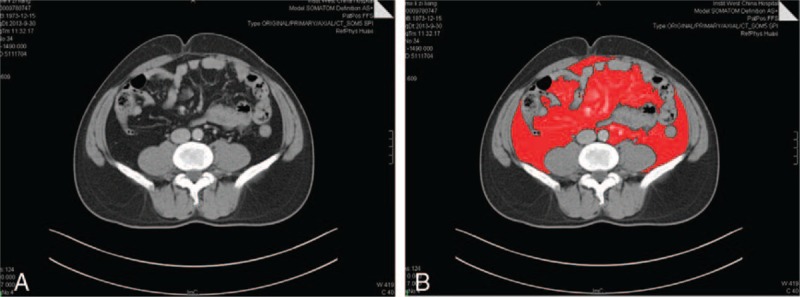
(A) Computed tomography image of umbilicus level and (B) visceral fat area which was measured by Analyze 11.0.

1. Body mass index (BMI): In western research, BMI ≥30 kg/m^2^ was defined obesity according to World Health Organization (WHO) classification.^[8,9]^ However, the percentage of population with BMI ≥30 kg/m^2^ is no more than 2.0% to 3.0% in Japan and is 10% to 20% in Europe and America.^[10–13]^ There were only 13 (4%) patients with BMI ≥30 kg/m^2^ in our study. Furthermore, it has been reported that BMI was not always consistent with visceral fat area.^[11,14]^ The percentage of fat volume in Asians is 3% to 5% higher than that in Europeans and Americans for the same BMI.^[15]^ Asians tend to accumulate visceral fat.^[16]^ Therefore, the definition of obesity by BMI is inevitably different among various ethnic groups.^[14,17]^ The WHO Steering Committee of the Western Pacific Region has proposed the definition of obesity as BMI ≥25 kg/m^2^ for the Asia-Pacific region, which is lower than the WHO classification.^[16]^ Obesity in Japan is adequately specified as BMI ≥25 kg/m^2^ by the Japan Society for the Study of Obesity.^[11]^ Based on this criterion, BMI ≥25 kg/m^2^ was defined obesity in our study. The same classification was also used by other studies.^[18,19]^
2. Visceral fat area (VFA): CT image of umbilicus level was acquired and analyzed by Analyze 11.0. VFA was measured by calculating ROI (region of interest) area,^[19–21]^ as shown in Fig. 1A and B. All measurements were under the surveillance of imaging doctors. VFA ≥100 cm^2^ was defined visceral obesity based on the criteria of Japan society for the study of obesity.^[11]^
3. Visceral fat area/body surface area (VFA/BSA): VFA/BSA ≥85 cm^2^/m^2^ was defined obesity according to literature.^[22]^
4. Mesorectum fat ratio (MFR): Intraabdominal fat area/intraabdominal area.
5. Pelvic fat area (PFA): PFA was measured at the acetabulum level using Analyze 11.0.
6. Pelvic fat ratio (PFR): Intrapelvic fat area/intrapelvic area.

### 2.4 Evaluation of completion of mesorectum

Based on the standard proposed by Quirke et al,^[7]^ we made some adjustments:

- Grade 3 (Complete): Intact mesorectum with only minor irregularities of a smooth mesorectal surface. No defect deeper than 5 mm, and no coning toward the distal margin of the specimen. There is a smooth circumferential resection margin (CRM) on slicing
  (3a) the mesorectum is complete without any defection of facia propria
  (3b) there have defection of fascia propria on the mesorectum, the number of defections is no more than 2, and the area of the defection is less than 2 cm × 2 cm
  (3c) defections of fascia propria are larger or more than 3b with depth less than 5 mm.
- Grade 2 (Nearly complete): moderate bulk to the mesorectum, but irregularity of the mesorectal surface. Moderate coning of the specimen is allowed. At no site is the muscularis propria visible, with the exception of the insertion of the levator muscles
- Grade 1 (Incomplete): little bulk to mesorectum with defects down onto muscularis propria and/or very irregular CRM
  (1a) muscular propria can be seen anteriorly;
  (1b) muscular propria can be seen bilaterally;
  (1c) muscular propria can be seen posteriorly.

To be specific, 3a was defined as complete mesorectum; 3b and 3c were defined as subcomplete mesorectum; 2, 1a, 1b, and 1c were defined as incomplete mesorectum. In this study, we made some adjustments: subcomplete and incomplete mesorectum were both regarded as incomplete resection. Pathologist evaluated every specimen under the guidance of the modified standard.

### 2.5 Surgical procedure

Patients were placed in the lithotomy position. Straight laparoscopic surgeries were performed under the principal of tumor free and TME. Four ports were used for this procedure with the surgeon standing on the right side of the patient. The sigmoid colon was mobilized using a media to lateral method. The inferior mesenteric artery was usually divided at the level of about 1 to 1.5 cm above its origin from the aorta. The descending colon was also mobilized upward to the splenic flexure. The pelvic mobilization of the rectum was performed with a harmonic scalpel downward to the levator level. The dissection plain should stay closely to the visceral layer of pelvic fascia posteriorly and laterally and anterior to the posterior layer of Denovilliers’ fascia, so as to avoid dissecting into the envelope of mesorectum and to preserve the autonomic nerves. The distance from the tumor to distal margin was more than 2 to 5 cm, and to the proximal margin was more than 15 cm. Then, a small incision was made by extending the left port for taking out the specimen.

### 2.6 Perioperative care

Laparoscopic TME was performed at least 6 weeks after completion of radiotherapy and/or chemotherapy. Preoperative mechanical bowel preparation was performed. The duration of preoperative fasting was 2 hours for liquids and 6 to 8 hours for solids. During induction to anesthesia, they received a second-generation cephalosporin (cefoxitin), 1 g intravenously and an additional dose was administered every 3 hours during surgery, and 1 dose of cefoxitin was administered every 12 hours within postoperative day 3. Low-dose heparin was given by subcutaneous injection for prophylaxis of thromboembolism until the first postoperative ambulation. Water could be taken orally on the day of surgery. Feeding began after the passage of flatus and started with a low-residue diet, progressing to a regular diet on the following day.

### 2.7 Statistical analysis

Collected data were analyzed by SPSS 20.0. Dichotomous variables were analyzed using Chi-square or Fisher exact test. Continuous variables matching normal distribution were showed in mean ± standard deviation and analyzed using Student *t* test; otherwise were showed in median (interquartile range) format and analyzed using nonparametric test. *P* < 0.05 was considered to be statistically significant.

## 3 Results

From July 2011 to April 2014, 424 consecutive patients who underwent rectal surgery were enrolled in the study after reviewing patient board. Eighty-seven patients underwent open surgery, 6 underwent transanal resection, and 9 converted to open surgery were excluded. Finally, 322 patients underwent laparoscopic rectal surgery were included. Population characteristics are shown in Table 1.

**Table 1:**
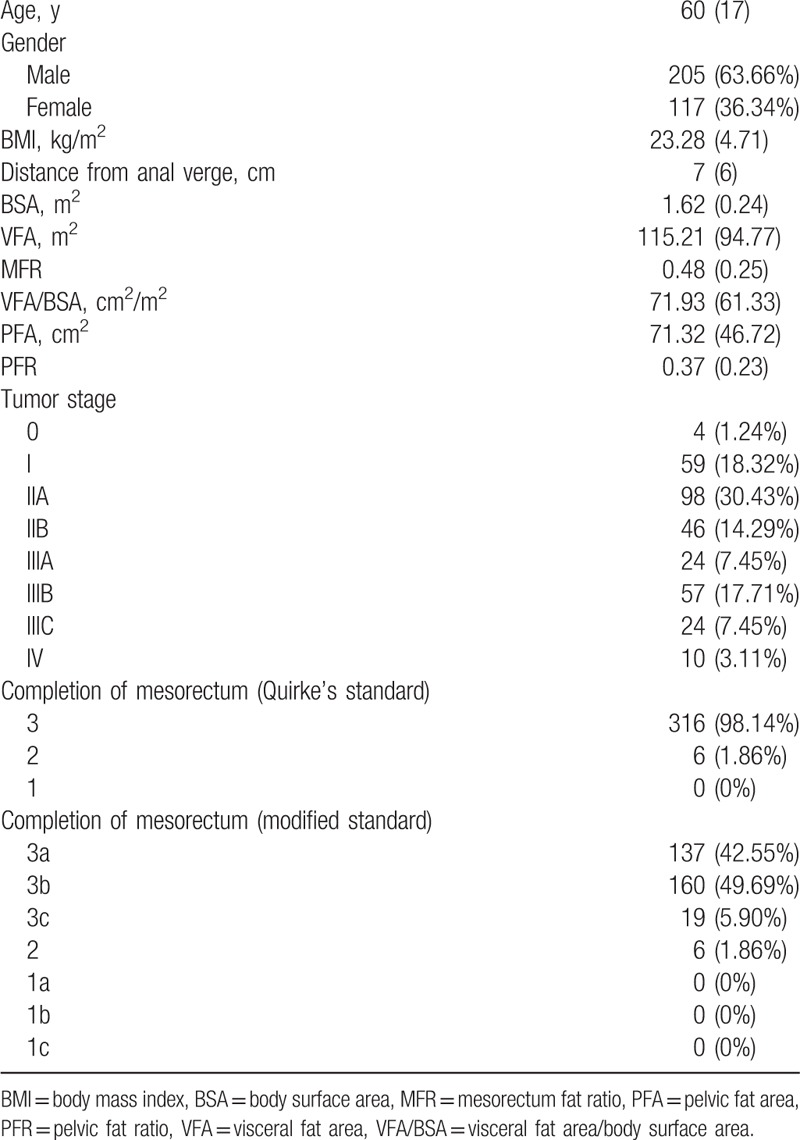
Population characteristics.

### 3.1 Completion of mesorectum

Patients’ specimens were classified by Quirke's standard and our modified standard and data are shown in Table 1. In this study, subcomplete and incomplete mesorectum were both regarded as incomplete resection and combined to undergo statistical analysis.

### 3.2 Impact of obesity on outcomes

In BMI groups, no significant differences were found in surgical quality and outcome between the 2 groups (*P* ≥ 0.05). However, in VFA groups, completion of mesorectum (*P* = 0.002), operative time (*P* = 0.02), and incision length (*P* = 0.02) were significantly different between the 2 groups. In VFA/BSA groups, completion of mesorectum (*P* = 0.002) and incision length (*P* = 0.009) were significantly different between the 2 groups. Data are shown in Table 2.

**Table 2:**
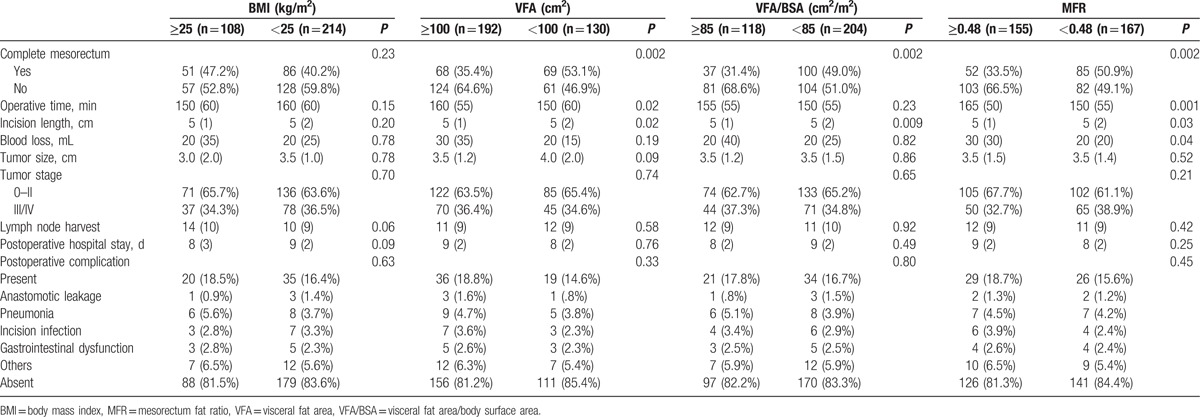
Impact of body mass index, visceral fat area, visceral fat area/body surface area, and mesorectum fat ratio on laparoscopic rectal surgery.

### 3.3 Impact of MFR on outcomes

Since no cut-off line was reported based on MFR, we applied upper quartile (0.59), median number (0.48), and lower quartile (0.34) as cutoff line to analyze (see Supplementary Table 1 which shows the impact of mesorectum fat ratio on laparoscopic rectal surgery). When MFR was equal to 0.48, completion of mesorectum (*P* = 0.002), operative time (*P* = 0.001), incision length (*P* = 0.03), and blood loss (*P* = 0.04) were significantly different between the 2 groups. Hence, we decided to set the cutoff line at 0.48. MFR ≥ 0.48 was defined as higher MFR and MFR < 0.48 was defined as lower MFR. Data are shown in Table 2.

In respect to morbidity, 29 patients in higher MFR group developed postoperative complication. Four had anastomotic leakage, 11 had pulmonary infection, 6 had wound infection, 3 had ileus and 5 had urinary retention. Twenty-six patients in lower MFR group developed postoperative complication. One had anastomotic leakage, 8 had pulmonary infection, 9 had wound infection, 4 had ileus, 2 had urinary retention, 1 had chylous fistulas, and 1 had bowel bacteria disorder. No significant difference was found between the 2 groups (*P* ≥ 0.05).

### 3.4 Impact of PFA and PFR on outcomes

In PFA and PFR groups, we applied upper quartile, median number, and lower quartile as cutoff line to analyze. There were no significantly differences between the 2 groups (*P* ≥ 0.05). (Data are shown in Supplementary Table 2 which shows the impact of PFA on laparoscopic rectal surgery; Supplementary Table 3 which shows the impact of PFR on laparoscopic rectal surgery.)

### 3.5 Analysis on data divided by gender

Male patients were analyzed separate from females. In BMI groups, no significant differences were found (*P* ≥ 0.05). In VFA groups, completion of mesorectum (*P* = 0.03) and operative time (*P* = 0.008) were significantly different between the 2 groups. In VFA/BSA groups, completion of mesorectum (*P* = 0.04) and incision length (*P* = 0.02) were significantly different between the 2 groups. In MFR groups, completion of mesorectum (*P* = 0.02) and operative time (*P* = 0.001) were significantly different between the 2 groups (see Supplementary Table 4 which shows the impact of body mass index, visceral fat area, visceral fat area/body surface area, and mesorectum fat ratio on laparoscopic rectal surgery in male). When the data of female patients were analyzed, no significant differences were found in BMI groups (*P* ≥ 0.05). In VFA, VFA/BSA, and MFR group, completion of mesorectum were significantly different between the 2 groups (*P* = 0.02, 0.003, and 0.045). Data are shown in Supplementary Table 5 (which shows the impact of body mass index, visceral fat area, visceral fat area/body surface area, and mesorectum fat ratio on laparoscopic rectal surgery in female).

### 3.6 Logistic regression model

After the analysis of logistic regression, only obesity defined with VFA was the risk factor of incomplete mesorectum excision (*P* = 0.001, Table 3).

**Table 3:**
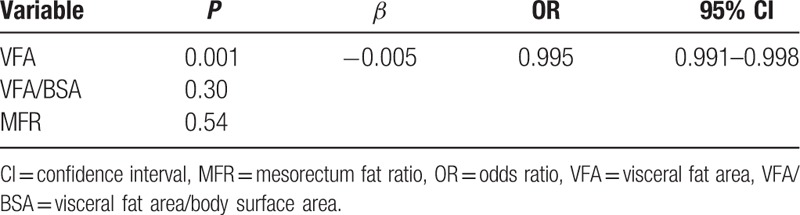
Multivariable logistic regression model.

## 4 Discussion

In 1982, Heald came up with the concept of TME and greatly improved prognostic outcome of rectal cancer.^[2]^ Mesorectum is the adipose connective tissue wrapped by the rectal proper fascia. Cancerous node could be located in anywhere within 4 cm proximal to primary tumor. Residual of mesorectum is one of the main factors to determine local recurrence. Residual of mesorectal adipose tissue is one of the causes to local recurrence after rectal surgery. Nagtegaal et al^[23]^ found that patients with complete mesorectal excision had significant lower rate of local recurrence than patients with incomplete mesorectal excision. Quirke et al^[3]^ further confirmed the result and developed the standard to evaluate completion of mesorectum. The randomized controlled trial also demonstrated that the rates of local recurrence of good and poor completion of mesorectum were 13% and 7%.^[3]^ Quirke's standard has been accepted by more and more surgeons and used to control quality of surgery.^[3,24,25]^ In our study, 316 out of 322 (98.14%) patients underwent laparoscopic surgery received good completion of mesorectum. Gouvas et al^[26]^ also showed that laparoscopic procedure could achieve better rate of good completion of mesorectum than open procedure. For experienced surgeons, it is much easier to achieve good completion of mesorectum through laparoscopic surgery than open surgery. The impact of obesity on laparoscopic rectal surgery has been reduced since the increasing practice and experience of TME.^[27–29]^

When applying Quirke's standard, due to lack of cases in medium and poor completion of mesorectum, we failed to identify any difference in completion of mesorectum with no matter what index. Considering Quirke's standard is relatively rough and based on European population, we believe that this standard has some limitations. First, the standard only considers whether the depth of mesorectum defect is over 0.5 cm but without consideration of defect of proper fascia of rectum. Besides, the scale and location of mesorectum defect are also important. Second, the standard only examines whether the proximal specimen is a cone. Third, Asian populations, especially southern Asian populations, have relatively thinner body figure. Of whom the mesorectum are thin, especially the anterior mesorectum (measure at the middle of seminal vesicle). In the present study, the thickness of anterior mesorectum was 0.61 ± 0.16 cm. Defect over 0.5 cm means the area of residual mesorectum is extremely large. As a result, we modified the Quirke's standard to further specify the evaluation.

We found that BMI is not suitable for predicting surgical difficulties and outcomes. Similar results were found between BMI-obese patients and nonobese patients. Possible explanation to this phenomenon is that BMI could not reflect body fat distribution.^[11,14]^ Excessive amount of visceral fat is the genuine factor that influences surgical difficulty and outcome. Asian populations are reported to have lower BMI but higher proportion of intraabdominal fat (male: BMI 23.4 ± 3.0 kg/m^2^, fat proportion 21.4 ± 6.3%; female: BMI 22.5 ± 3.3 kg/m^2^, fat proportion 31.6 ± 6.5%).^[30]^ Some studies suggested that VFA or VFA/BSA should be used to measure the degree of visceral obesity.^[19,21,31,32]^ Japan society for the study of obesity even proposed that visceral obesity should be defined by VFA.^[11]^

Our study showed higher VFA was associated with longer operative time and longer length of incision. The completion of mesorectum was significantly lower when VFA was over 100 cm^2^. When the data of male patients were analyzed separate from females, the completion of mesorectum was significantly lower when VFA was over 100 cm^2^, as well as the result in female groups. The result of logistic regression also demonstrated that VFA was correlated to incomplete mesorectum. Our finding is in coordinate with previous studies. Conclusively, we believe VFA is a better index to reflect surgical quality and difficulty than BMI, MFR, and VFA/BSA. Colorectal surgeons may predict surgical difficulty and outcome by measuring VFA preoperatively. So far, no study has investigated the impact of VFA on the quality of surgery. Our study may be the first one to demonstrate that VFA is a sensitive index in evaluating the impact of visceral fat on rectal cancer surgery.^[31–33]^

Theoretically, VFA is merely the absolute value of intraabdominal fat area. Individuals share the same degree of visceral obesity may have different VFA because of different body figure. Thus, by taking body figure's influence into consideration, we introduced VFA/BSA and MFR. Our findings showed VFA/BSA was associated with length of incision and completion of mesorectum while MFR was associated with operative time, incision length, blood loss, and completion of mesorectum. However, multivariable regression showed both VFA/BSA and MFR were not risk factors of incomplete mesorectum. Although these 2 indexes are better in reflecting degree of visceral obesity, they are less sensitive indexes than VFA in predicting surgical difficulty and quality. One possible reason is that VFA has a larger dispersion (*R* = 475.07 cm^2^). At the same level of measurement, VFA remains relatively constant among individuals. Introducing body figure ratios may conversely reduce the difference among individuals. Small sample size of our study may also influence the interpretation of results.

Secondly, current studies prefer measuring VFA at umbilicus level or L3–L4 level, which has the best correlation to total visceral fat volume. There is no certain standard to demonstrate intrapelvic fat area. Since rectal surgery is mainly performed inside the pelvic cavity, we presumed that intrapelvic fat area might present a more direct index than VFA to reflect the impact of visceral obesity. However, we found no statistical difference between the 2 groups. The measurement of intrapelvic fat area may be affected by following factors: filling or empty bladder; the presence of uterus and its size and location; higher variation of measuring level (compared to umbilicus level). Therefore, a better index that can precisely reflect intrapelvic fat volume is required in future studies.

Surgical quality is crucial for prognosis after rectal cancer surgery. Completion of mesorectum and CRM are the top 2 indexes to affect local recurrence and long-term survival.^[34,35]^ The rate of local recurrence of T3 rectal cancer could be reduced because mesorectum residuals can be treated by neoadjuvant or adjuvant radiotherapy. The National Comprehensive Cancer Network guideline recommended routine use of neoadjuvant radiotherapy. Nevertheless, the guideline of ESMO did not recommend routine use of neoadjuvant radiotherapy for T3/CRM− patients due to the side effects to defecation, urinary, and sexual functions. Researchers suggested it should be not necessary to perform neoadjuvant or adjuvant radiotherapy on CRM negative cases according to preoperative evaluation. Ferenschild et al^[36]^ found that T2–3N0 patients did not benefit from neoadjuvant radiotherapy (local recurrence rate: 6% vs 6% *P* > 0.05). Taylor et al^[37]^ reported 5-year local recurrence rate was only 3% for T2/T3a/T3b and CRM negative patients without neoadjuvant radiotherapy. Predicting potential outcome of completion of mesorectum and CRM are of importance for making perioperative treatment plan. In our study, 24 out of 322 patients were found CRM positive (11 with complete mesorectum and 13 with incomplete mesorectum). The rate of CRM positive was comparable between the 2 groups. Hence, for patients without preoperative radiotherapy, adjuvant radiotherapy could be skipped if CRM is negative and there is no lymph node metastasis.

## 5 Conclusion

Evaluating completion of mesorectum has been widely adopted to control the quality of rectal cancer surgery. Laparoscopic surgery of rectal cancer can achieve better completion of mesorectum than open surgery. BMI does not reflect the impact of obesity on laparoscopic rectal surgery. When applying stricter standard to evaluate completion of mesorectum, VFA is a better index in predicting the influence of visceral obesity on surgical quality and difficulty of laparoscopic rectal surgery than VFA/BSA and MFR.

## Supplementary Material

Supplemental Digital Content
