# Supplementary material for: The impact of general/visceral obesity on completion of mesorectum and perioperative outcomes of laparoscopic TME for rectal cancer: A STARD-compliant article
Source: Medicine (Baltimore). 2016 Sep 9;95(36):e4462. doi: 10.1097/MD.0000000000004462 (PMC5023862; doi:10.1097/MD.0000000000004462)
Supplement: Supplemental Digital Content [file medi-95-e4462-s001.doc]

Article title: Which indexes of obesity reflect the better completion of mesorectum in laparoscopic rectal surgery? First author: Bingchen Chen

| Supplementary Tables 1 Impact of mesorectum fat ratio on laparoscopic rectal surgery | | | | | | | | | |  |
| --- | --- | --- | --- | --- | --- | --- | --- | --- | --- | --- |
|  | MFR | |  | MFR | |  | MFR | |  |  |
|  | ≥0.34  (n=238) | <0.34  (n=84) | p | ≥0.48  (n=155) | <0.48  (n=167) | p | ≥0.59  (n=77) | <0.59  (n=245) | p |  |
| Complete mesorectum |  |  | .002 |  |  | .002 |  |  | .32 |  |
| Yes | 89(37.4%) | 48(57.1%) |  | 52(33.5%) | 85(50.9%) |  | 29(37.7%) | 108(44.1%) |  |  |
| No | 149(62.6%) | 36(42.9%) |  | 103(66.5%) | 82(49.1%) |  | 48(62.3%) | 137(55.9%) |  |  |
| Operative time (min) | 160(60) | 150(55) | .05 | 165(50) | 150(55) | .001 | 170(63) | 150(50) | .03 |  |
| Incision length (cm) | 5(1) | 5(2) | .19 | 5(1) | 5(2) | .03 | 5(1) | 5(1) | .44 |  |
| Blood loss (ml) | 20(35) | 20(25) | .91 | 30(30) | 20(20) | .04 | 30(30) | 20(25) | .04 |  |
| Lymph node harvest | 11(9) | 12(11) | .25 | 12(9) | 11(9) | .42 | 12(10) | 11(10) |  |  |
| Postoperative hospital stay (d) | 9(2) | 8(2) | .25 | 9(2) | 8(2) | .25 | 9(3) | 9(2) | .67 |  |
| Postoperative complication |  |  | .65 |  |  | .45 |  |  | .18 |  |
| present | 42(17.6%) | 13(15.5%) |  | 29(18.7%) | 26(15.6%) |  | 17(22.1%) | 38(15.5%) |  |  |
| absent | 196(82.4%) | 71(84.5%) |  | 126(81.3%) | 141(84.4%) |  | 60(77.9%) | 207(84.5%) |  |  |

MFR = Mesorectum fat ratio

Article title: Which indexes of obesity reflect the better completion of mesorectum in laparoscopic rectal surgery? First author: Bingchen Chen

| Supplementary Tables 2 Impact of pelvic fat area on laparoscopic rectal surgery | | | | | | | | | |
| --- | --- | --- | --- | --- | --- | --- | --- | --- | --- |
|  | PFA (cm²) | |  | PFA (cm²) | |  | PFA (cm²) | |  |
|  | ≥51  (n=241) | <51  (n=81) | p | ≥71  (n=162) | <71  (n=160) | p | ≥97  (n=82) | <97  (n=240) | p |
| Complete mesorectum |  |  | .36 |  |  | .18 |  |  | .31 |
| Yes | 99(41.1%) | 38(46.9%) |  | 63(38.9%) | 74(46.2%) |  | 31(37.8%) | 106(44.2%) |  |
| No | 142(58.9%) | 43(53.1%) |  | 99(61.1%) | 86(53.8%) |  | 51(62.2%) | 134(55.8%) |  |
| Operative time (min) | 150(55) | 160(60) | .66 | 150(50) | 160(63) | .49 | 155(54) | 153(55) | .88 |
| Incision length (cm) | 5(1) | 5(1) | .67 | 5(1) | 5(1) | .89 | 5(1) | 5(1) | .23 |
| Blood loss (ml) | 20(33) | 20(40) | .78 | 20(25) | 20(35) | .91 | 30(20) | 20(40) | .33 |
| Lymph node harvest | 11(9) | 13(12) | .15 | 11(10) | 12(9) | .82 | 11(11) | 12(8) | .47 |
| Postoperative hospital stay (d) | 9(2) | 8(3) | .26 | 9(2) | 8(2) | .17 | 9(2) | 9(2) | .81 |
| Postoperative complication |  |  | .19 |  |  | .32 |  |  | .31 |
| present | 45(18.7%) | 10(12.3%) |  | 31(19.1%) | 24(15.0%) |  | 17(20.7%) | 38(15.8%) |  |
| absent | 196(81.3%) | 71(87.7%) |  | 131(80.9%) | 135(85.0%) |  | 65(79.3%) | 202(84.2%) |  |

PFA = Pelvic fat area

Article title: Which indexes of obesity reflect the better completion of mesorectum in laparoscopic rectal surgery? First author: Bingchen Chen

| Supplementary Tables 3 Impact of pelvic fat ratio on laparoscopic rectal surgery | | | | | | | | | |  |
| --- | --- | --- | --- | --- | --- | --- | --- | --- | --- | --- |
|  | PFR | |  | PFR | |  | PFR | |  |  |
|  | ≥0.28  (n=243) | <0.28  (n=79) | p | <0.37  (n=164) | <0.37  (n=158) | p | ≥0.51  (n=77) | <0.51  (n=245) | p |  |
| Complete mesorectum |  |  | .87 |  |  | .28 |  |  | .07 |  |
| Yes | 104(42.8%) | 33(41.8%) |  | 65(39.6%) | 72(45.6%) |  | 26(33.8%) | 111(45.3%) |  |  |
| No | 139(57.2%) | 46(58.2%) |  | 99(60.4%) | 86(54.4%) |  | 51(66.2%) | 134(54.7%) |  |  |
| Operative time (min) | 155(53) | 155(68) | .37 | 153(54) | 155(65) | .94 | 160(53) | 150(55) | .48 |  |
| Incision length (cm) | 5(1) | 5(1) | .11 | 5(1) | 5(1) | .48 | 5(1) | 5(2) | .11 |  |
| Blood loss (ml) | 25(35) | 20(38) | .14 | 20(30) | 20(35) | .89 | 20(30) | 20(40) | .77 |  |
| Lymph node harvest | 12(9) | 11(10) | .57 | 11(8) | 12(10) | .82 | 11(9) | 12(9) | .64 |  |
| Postoperative hospital stay (d) | 9(2) | 8(3) | .11 | 9(2) | 8(2) | .18 | 9(2) | 9(2) | .57 |  |
| Postoperative complication |  |  | .06 |  |  | .06 |  |  | .52 |  |
| present | 47(19.3%) | 8(10.1%) |  | 35(21.3%) | 20(12.7%) |  | 15(19.5%) | 40(16.3%) |  |  |
| absent | 196(80.7%) | 71(89.9%) |  | 129(78.7%) | 138(87.3%) |  | 62(80.5%) | 205(83.7%) |  |  |

PFR = Pelvic fat ratio

| Article title: Which indexes of obesity reflect the better completion of mesorectum in laparoscopic rectal surgery? First author: Bingchen Chen  Supplementary Tables 4 Male: impact of body mass index, visceral fat area, visceral fat area/body surface area and mesorectum fat ratio on laparoscopic rectal surgery | | | | | | | | | | | | |
| --- | --- | --- | --- | --- | --- | --- | --- | --- | --- | --- | --- | --- |
|  | BMI ^a^ (Kg/m²) | |  | VFA ^b^ (cm²) | |  | VFA/BSA ^c^ (cm²/m^2^) | |  | MFR ^d^ | |  |
|  | ≥25  (n=67) | <25  (n=138) | P | ≥100  (n=126) | <100  (n=79) | P | ≥85  (n=72) | <85  (n=133) | P | ≥0.48  (n=98) | <0.48  (n=107) | p |
| Complete mesorectum |  |  | .44 |  |  | .03 |  |  | .04 |  |  | .02 |
| Yes | 31(46.3%) | 56(40.6%) |  | 46(36.5%) | 41(50.6%) |  | 25(34.7%) | 62(46.6%) |  | 33(33.7%) | 54(50.5%) |  |
| No | 36(53.7%) | 82(59.4%) |  | 80(63.5%) | 38(49.4%) |  | 47(65.3%) | 71(53.4%) |  | 65(66.3%) | 53(49.5%) |  |
| Operative time (min) | 155(60) | 160(55) | .18 | 170(54) | 150(56) | .008 | 170(55) | 155(55) | .16 | 175(50) | 150(55) | .001 |
| Incision length (cm) | 5(1) | 5(2) | .05 | 5(1) | 5(2) | .10 | 5(1) | 5(2) | .02 | 5(1) | 5(2) | .10 |
| Blood loss (ml) | 30(31) | 25(30) | .53 | 30(30) | 20(25) | .21 | 30(35) | 25(30) | .62 | 30(30) | 20(35) | .26 |
| Lymph node harvest | 14(11) | 10(8) | .05 | 11(10) | 11(8) | .72 | 12(9) | 11(9) | .79 | 11(10) | 11(10) | .66 |
| Postoperative hospital stay (d) | 8(2) | 9(2) | .15 | 9(2) | 8(2) | .89 | 8(3) | 9(2) | .31 | 9(2) | 9(2) | .50 |
| Postoperative complication |  |  | .56 |  |  | .08 |  |  | .58 |  |  | .50 |
| Present | 16(23.9%) | 28(20.3%) |  | 32(25.4%) | 12(15.2%) |  | 17(23.6%) | 27(20.3%) |  | 23(23.5%) | 21(19.6%) |  |
| Absent | 51(76.1%) | 110(79.7%) |  | 94(74.6%) | 67(84.8%) |  | 55(76.4%) | 106(79.7%) |  | 75(76.5%) | 86(80.4%) |  |

BMI = body mass index; VFA = visceral fat area; VFA/BSA = visceral fat area/body surface area; MFR = mesorectum fat ratio

| Article title: Which indexes of obesity reflect the better completion of mesorectum in laparoscopic rectal surgery? First author: Bingchen Chen  Supplementary Tables 5 female: impact of body mass index, visceral fat area, visceral fat area/body surface area and mesorectum fat ratio on laparoscopic rectal surgery | | | | | | | | | | | | | |
| --- | --- | --- | --- | --- | --- | --- | --- | --- | --- | --- | --- | --- | --- |
|  | | BMI ^a^ (Kg/m²) | |  | VFA ^b^ (cm²) | |  | VFA/BSA ^c^ (cm²/m^2^) | |  | MFR ^d^ | |  |
|  |  | ≥25  (n=41) | <25  (n=76) | P | ≥100  (n=66) | <100  (n=51) | P | ≥85  (n=46) | <85  (n=71) | P | ≥0.48  (n=57) | <0.48  (n=60) | p |
| Complete mesorectum | |  |  | .56 |  |  | .02 |  |  | .003 |  |  | .045 |
| Yes | 19(46.3%) | | 31(40.8%) |  | 22(33.3%) | 28(54.9%) |  | 12(26.1%) | 38(53.5%) |  | 19(33.3%) | 31(51.7%) |  |
| No | 22(53.7%) | | 45(59.2%) |  | 44(66.7%) | 23(45.1%) |  | 34(73.9%) | 33(46.5%) |  | 38(66.7%) | 29(48.3%) |  |
| Operative time (min) | 150(55) | | 150(60) | .73 | 150(55) | 150(60) | .77 | 150(50) | 150(60) | .18 | 150(50) | 150(55) | .70 |
| Incision length (cm) | 5(1) | | 5(2) | .56 | 5(1) | 5(1) | .15 | 5(1) | 5(1) | .22 | 5(1) | 5(1) | .18 |
| Blood loss (ml) | 20(40) | | 20(20) | .88 | 20(30) | 20(20) | .80 | 20(33) | 20(20) | .07 | 20(30) | 20(20) | .92 |
| Lymph node harvest | 14(9) | | 11(9) | .20 | 11(9) | 14(10) | .18 | 12(8) | 12(12) | .38 | 12(8) | 12(10) | .56 |
| Postoperative hospital stay (d) | 8(2) | | 9(2) | .36 | 9(2) | 8(2) | .80 | 9(2) | 8(2) | .27 | 8(2) | 9(2) | .80 |
| Postoperative complication |  | |  | .92 |  |  | .16 |  |  | .83 |  |  | .69 |
| Present | 4(9.8%) | | 7(9.2%) |  | 4(6.1%) | 7(13.7%) |  | 4(8.7%) | 7(9.9%) |  | 6(10.5%) | 5(8.3%) |  |
| Absent | 37(90.2%) | | 69(90.8%) |  | 62(93.9%) | 44(86.3%) |  | 42(91.8%) | 64(90.1%) |  | 51(89.5%) | 55(91.7%) |  |

BMI = body mass index; VFA = visceral fat area; VFA/BSA = visceral fat area/body surface area; MFR = mesorectum fat ratio
